# UbcH10 overexpression may represent a marker of anaplastic thyroid carcinomas

**DOI:** 10.1038/sj.bjc.6602721

**Published:** 2005-08-02

**Authors:** P Pallante, M T Berlingieri, G Troncone, M Kruhoffer, T F Orntoft, G Viglietto, A Caleo, I Migliaccio, M Decaussin-Petrucci, M Santoro, L Palombini, A Fusco

**Affiliations:** 1 Dipartimento di Biologia e Patologia Cellulare e Molecolare c/o Istituto di Endocrinologia ed Oncologia Sperimentale del CNR, Facoltà di Medicina e Chirurgia di Napoli, Università degli Studi di Napoli ‘Federico II’, via Pansini, 5, 80131 Naples, Italy; 2 Dipartimento di Anatomia Patologica e Citopatologia, Facoltà di Medicina e Chirurgia di Napoli, Università di Napoli ‘Federico II’, via Pansini, 5, 80131 Naples, Italy; 3 Department of Clinical Biochemistry, Aarhus University Hospital, Skejby DK 8200 Aarhus N, Denmark; 4 Service d’Anatomo-Pathologie, Centre Hospitalier Lyon Sud, Pierre Bénite, France; 5 NOGEC (Naples Oncogenomic Center)-CEINGE, Biotecnologie Avanzate, via Comunale Margherita, 80131 Naples, Italy

**Keywords:** UbcH10, thyroid, carcinomas, immunohistochemistry

## Abstract

The hybridisation of an Affymetrix HG_U95Av2 oligonucleotide array with RNAs extracted from six human thyroid carcinoma cell lines and a normal human thyroid primary cell culture led us to the identification of the UbcH10 gene that was upregulated by 150-fold in all of the carcinoma cell lines in comparison to the primary culture cells of human normal thyroid origin. Immunohistochemical studies performed on paraffin-embedded tissue sections showed abundant UbcH10 levels in thyroid anaplastic carcinoma samples, whereas no detectable UbcH10 expression was observed in normal thyroid tissues, in adenomas and goiters. Papillary and follicular carcinomas were only weakly positive. These results were further confirmed by RT–PCR and Western blot analyses. The block of UbcH10 protein synthesis induced by RNA interference significantly reduced the growth rate of thyroid carcinoma cell lines. Taken together, these results would indicate that UbcH10 overexpression is involved in thyroid cell proliferation, and may represent a marker of thyroid anaplastic carcinomas.

Tumours are the result of the accumulation of different modifications in critical genes involved in the control of cell proliferation. In a large number of carcinomas with worst prognosis, lesions are not diagnosed until the disease is at an advanced stage. Although various therapeutic approaches are followed in clinical practice, most of them are not life saving. Hence, the discovery of ways to diagnose cancer at an early stage and to establish more effective therapies is a critical and urgent issue. To achieve this goal, identification and characterisation of key molecules that participate in carcinogenesis are essential steps. Thyroid neoplasms represent a good model for studying the events involved in epithelial cell multistep carcinogenesis, because they comprise a broad spectrum of lesions with different degrees of malignancy from benign adenomas, which are not invasive and very well differentiated, to the undifferentiated anaplastic thyroid carcinomas, which are very aggressive and always fatal; papillary and follicular carcinomas, the most common forms of thyroid cancer, represent intermediate forms of neoplasia being differentiated and having a good prognosis (Hedinger *et al*, 1989; Wynford-Thomas, 1997).

The involvement of several oncogenes has been demonstrated in papillary thyroid carcinomas. Activation of the RET/PTC oncogene, caused by rearrangements of the RET protooncogene, is detectable in about 30% of the cases (Grieco *et al*, 1990; Tallini *et al*, 1998). Mutations of the B-RAF gene have been demonstrated in almost 40% of papillary carcinomas (Fukushima *et al*, 2003). TRK gene rearrangements (Pierotti *et al*, 1995) and MET gene overexpression are often found in papillary carcinomas (Di Renzo *et al*, 1992). RAS gene mutations (Suarez *et al*, 1990) and PAX8-PPAR-*γ* rearrangements (Kroll *et al*, 2000) are frequently detected in tumours of the follicular type. Impairment of the p53 tumour suppressor gene function represents a typical feature of the anaplastic carcinomas (Ito *et al*, 1992; Dobashi *et al*, 1993; Donghi *et al*, 1993; Fagin *et al*, 1993; Matias-Guiu *et al*, 1994). Even though critical molecular mechanisms of thyroid carcinogenesis have been clarified, other molecular steps of neoplastic progression need to be investigated.

Therefore, to identify the genes regulated in the process of thyroid carcinogenesis, we analysed a microarray with RNAs extracted from normal human thyroid primary cell culture (NTPC), and six human thyroid carcinoma cell lines of different histotype (one from a follicular carcinoma, three derived from papillary carcinomas and two from anaplastic carcinomas). Our attention was focused on the UbcH10 gene that was upregulated about 150-fold in all of the cell lines tested by the cDNA microarray. The UbcH10 gene belongs to the E2 gene family and codes for a protein of 19.6 kDa that is involved in the ubiquitin-dependent proteolysis. In this pathway, ubiquitin-conjugating enzyme (E2), together with ubiquitin ligase (E3), transfers ubiquitin to specific substrate proteins (Hershko and Ciechanover, 1998; Joazeiro and Weissman, 2000).

The expression level of UbcH10 was extremely low in the normal thyroid primary culture cells, but strong in all the cancerous cell lines. Immunohistochemical and RT–PCR analyses on a large panel of thyroid neoplasms of different histotypes revealed an increased UbcH10 expression in anaplastic thyroid carcinomas, whereas follicular and papillary carcinomas were just weakly positive. The block of UbcH10 protein synthesis by RNA interference inhibited the growth of two thyroid carcinoma cell lines.

## MATERIALS AND METHODS

### Cell culture and transfections

The human thyroid carcinoma cell lines used in this study are: TPC-1 (Tanaka *et al*, 1987), WRO (Estour *et al*, 1989), NPA and ARO (Pang *et al*, 1989), FRO (Fagin *et al*, 1993), NIM 1 (Zeki *et al*, 1993), B-CPAP (Fabien *et al*, 1994), FB-1 (Fiore *et al*, 1997), FB-2 (Basolo *et al*, 2002), Kat-4 and Kat-18 (Ain *et al*, 1997). They were grown in DMEM (Gibco Laboratories, Carlsbad, CA, USA) containing 10% fetal calf serum (Gibco Laboratories), glutamine (Gibco Laboratories) and ampicillin/streptomycin (Gibco Laboratories) in a 5% CO_2_ atmosphere. Normal human thyroid primary culture cells have been established and grown as already described (Curcio *et al*, 1994). PC Cl 3 cell line derived from Fischer rat thyroid (Fusco *et al*, 1987) was cultured in modified F12 medium supplemented with 5% calf serum (Gibco Laboratories) and six growth factors (thyrotropic hormone, hydrocortisone, insulin, transferrin, somatostatin and glycyl-histidyl-lysine; Sigma, St Louis, MO, USA). Thyroid cells were transfected using Lipofectamine reagent (Invitrogen, Carlsbad, CA, USA) according to the manufacturer's instructions. The transfected cells were selected in a medium containing geneticin (G418) (Life Technologies, Italy). For each transfection, several G418-resistant clones and the mass cell population were isolated and expanded for further analysis.

### Human thyroid tissue samples

Neoplastic human thyroid tissues and normal adjacent tissue or the controlateral normal thyroid lobe were obtained from surgical specimens and immediately frozen in liquid nitrogen. Thyroid tumours were collected at the Service d’Anatomo-Pathologie, Centre Hospitalier Lyon Sud, Pierre Bénite, France. The tumour samples were stored frozen until RNA or protein extractions were performed.

### RNA isolation

Total RNA was extracted from tissues and cell cultures using the RNAeasy mini kit (Qiagen, Valencia, CA, USA) according to the manufacturer's instructions. The integrity of the RNA was assessed by denaturing agarose gel electrophoresis.

### Reverse transcriptase–PCR analysis

In total, 5 *μ*g of total RNA from each sample, digested with DNAseI (Invitrogen), were reverse transcribed using random hexanucleotides and MuLV reverse transcriptase (Applied Biosystems, Foster City, CA, USA). PCR was carried out on cDNA using the GeneAmp PCR System 9600 (Applied Biosystems). RNA PCR Core Kit (Applied Biosystems) was used to perform RT–PCR reactions. For the UbcH10 gene, after a first denaturing step (94°C for 3 min), PCR amplification was performed for 25 cycles (94°C for 30 s, 57°C for 30 s, 72°C for 30 s). The sequences of forward and reverse primers, amplifying a fragment of 115 bp in the UbcH10 cDNA, were: forward 5′-GCCCGTAAAGGAGCTGAG-3′ and reverse 5′-GGGAAGGCAGAAATCCCT-3′. The human *β*-actin gene primers, amplifying a 109 bp cDNA fragment, were used as control; amplification was performed for 25 cycles (94°C for 30 s, 55°C for 30 s, 72°C for 30 s). *β*-Actin-forward, 5′-TCGTGCGTGACATTAAGGAG-3′; *β*-actin-reverse, 5′-GTCAGGCAGCTCGTAGCTCT-3′. To ensure that RNA samples were not contaminated with DNA, negative controls were obtained by performing the PCR on samples that were not reverse-transcribed, but otherwise identically processed. The PCR products were separated on a 2% agarose gel, stained with ethidium bromide and scanned using a Typhoon 9200 scanner. Quantitative PCR was performed in triplicate using iCycler (Bio-Rad, Hercules, CA, USA) with SYBR® Green PCR Master Mix (Applied Biosystems) as follows: 95°C 10 min and 40 cycles (95°C 15 s and 60°C 1 min). Fold mRNA overexpression was calculated according to the formula 2^(Rt−Et)^/2^(Rn−En)^ as described previously (El-Rifai *et al*, 2001), where Rt is the threshold cycle number for the reference gene in the tumour, Et for the experimental gene in the tumor, Rn for the reference gene in the normal sample and En for the experimental gene in the normal sample.

### Protein extraction, Western blotting and antibodies

Cells were washed once in cold PBS and lysed in a lysis buffer containing 50 mM HEPES, 150 mM NaCl, 1 mM EDTA, 1 mM EGTA, 10% glycerol, 1% Triton-X-100, 1 mM phenylmethylsulphonyl fluoride, 1 *μ*g aprotinin, 0.5 mM sodium orthovanadate, 20 mM sodium pyrophosphate. The lysates were clarified by centrifugation at 14 000 r.p.m. × 10 min. Protein concentrations were estimated by a Bio-Rad assay (Bio-Rad), and boiled in Laemmli buffer (Tris-HCl pH 6.8, 0.125 M, SDS 4%, glycerol 20%, 2-mercaptoethanol 10%, bromophenol blue 0.002%) for 5 min before electrophoresis. Proteins were subjected to SDS–PAGE (15% polyacrylamide) under reducing condition. After electrophoresis, proteins were transferred to nitrocellulose membranes (Immobilon-P Millipore Corp., Bedford, MA, USA); complete transfer was assessed using prestained protein standards (Bio-Rad). After blocking with TBS-BSA (25 mM Tris, pH 7.4, 200 mM NaCl, 5% bovine serum albumin), the membrane was incubated with the primary antibody against UbcH10 (Boston Biochem Inc., Cambridge, MA, USA) for 60 min (at room temperature). Primary antibody against c-Fos protein (Santa Cruz Biotechnology Inc., Santa Cruz, CA, USA) was used to confirm the specificity of siRNAs against UbcH10 protein. To ascertain that equal amounts of protein were loaded, the Western blots were incubated with antibodies against the *γ*-tubulin protein (Sigma). Membranes were then incubated with the horseradish peroxidase-conjugated secondary antibody (1 : 3000) for 60 min (at room temperature) and the reaction was detected with a Western blotting detection system (ECL; Amersham Biosciences, UK).

### Immunohistochemistry: tissue samples

UbcH10 protein cellular distribution was assessed by immunohistochemical analysis and compared to that of the standard cell proliferation marker Ki-67/MIB1. A series of surgical specimens from patients with thyroid diseases comprised of Hashimoto's thyroiditis-HT (six cases), nodular goiter (12 cases), follicular carcinoma (13 cases), papillary carcinoma (33 cases), poorly differentiated carcinoma (five cases) and anaplastic carcinoma (15 cases) was chosen to represent a wide range of thyroid pathology. As control, 10 areas of normal thyroid parenchyma were selected from the lobe controlateral to the tumour in surgical specimens of papillary carcinoma.

### Immunostaining: technique, evaluation and statistical analysis

Xylene dewaxed and alcohol-rehydrated paraffin sections were placed in Coplin jars filled with a 0.01 M tri-sodium citrate solution, and heated for 3 min in a conventional pressure cooker (Troncone *et al*, 2003). After heating, slides were thoroughly rinsed in cool running water for 5 min. They were then washed in Tris-Buffered Saline (TBS) pH 7.4 before incubating overnight with the specific antibody, diluted as follows: rabbit polyclonal *α*-UbcH10 (Boston Biochem) 1 : 1000; *α*-MIB-1 (Novocastra, Newcastle upon Tyne, UK) 1 : 50. After incubation with the primary antibody, tissue sections were stained with biotinylated anti-rabbit or anti-mouse immunoglobulins, followed by peroxidase-labelled streptavidine (Dako, Carpinteria, CA, USA); the signal was developed by using diaminobenzidine (DAB) chromogen as substrate. Incubations both omitting the specific antibody, and including unrelated antibodies, were used as negative controls.

Individual cells were scored for expression of UbcH10 and Ki-67 in similar areas of adjacent sections by quantitative analysis performed with a computerised analyser system (Ibas 2000, Kontron, Zeiss), as already described (Troncone *et al*, 2003). In each case, the distribution of these proteins was evaluated in at least 500 epithelial follicular cells and expressed as a percentage of the total cell population. The statistical analysis was performed using SPSS ^‘^Ver. 9.0.1 for Windows’. Data are expressed as median value and range. The nonparametric Mann–Whitney *U*-test was used to compare differences in labelling indexes for UbcH10 and Ki-67 in thyroid carcinomas. The Spearman rank order correlation was used to verify the association between UbcH10 and Ki-67. A *P*-value less than 0.05 was considered statistically significant.

### RNA interference

For small interfering RNA (siRNA) experiments, the following double-strand RNA oligo specific for UbcH10 coding region was used: 5′-AACCTGCAAGAAACCTACTCA-3′ as previously described (Wagner *et al*, 2004). As negative control, we used a corresponding scrambled sequence as follows: 5′-AACTAACACTAGCTCAAGACC-3′. All of the siRNA duplexes were purchased from Qiagen and were transfected using Oligofectamine (Invitrogen) according to the manufacturer's recommendations. Small interfering RNAs were used at a final concentration of 100 nM and 12 × 10^5^ cells well^−1^ were plated in a six-well format plates. Proteins were extracted 48 h after siRNA treatment and the levels of the UbcH10 protein were evaluated by Western blot.

### Assay of the transformed state

Tumorigenicity of the cell lines was tested by injecting 2 × 10^6^ cells subcutaneously into athymic mice. Soft agar colony assay was performed as previously described (Macpherson and Montagnier, 1964).

## RESULTS

### Expression of UbcH10 gene in normal human thyroid cells and thyroid carcinoma cell lines

**Figure 1:**
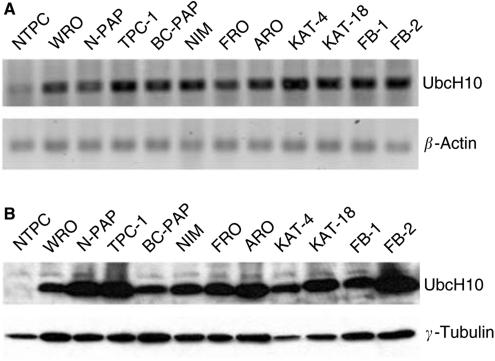
UbcH10 expression in human thyroid carcinoma cell lines. (**A**) UbcH10 gene expression analysis by RT–PCR in human thyroid carcinoma cell lines *vs* the normal human thyroid primary culture cells (NTPC). *β*-Actin gene expression was evaluated as control to normalise the amount of the used RNAs. (**B**) UbcH10 protein expression analysis by Western Blot in human thyroid carcinoma cell lines. Blot against *γ*-tubulin has been performed as control for equal protein loading.

To search for candidate genes involved in the neoplastic transformation of thyroid gland, RNAs extracted from normal human thyroid primary cells and six human thyroid carcinoma cell lines of different origin (WRO cell line from a follicular carcinoma, TPC-1 and FB-2 cell lines, both deriving from papillary thyroid cancers, NPA cell line, which derives from a poorly differentiated papillary carcinoma, ARO and FRO cell lines originating from anaplastic carcinomas) were hybridised to U95Av2 Affymetrix oligonucleotide arrays (data not shown). We concentrated our attention on the UbcH10 gene that was upregulated about 150-fold in all of the cell lines tested by the cDNA microarray. This result was confirmed by RT–PCR in a larger panel of thyroid carcinoma cell lines using as control normal thyroid primary culture (Figure 1A). Western blot analysis of UbcH10 expression, shown in Figure 1B, confirmed the RT–PCR data. In fact, the UbcH10 protein was abundantly expressed in all of the carcinoma cell lines, whereas it was barely detectable in normal thyroid cells.

### Analysis of UbcH10 expression in normal and neoplastic thyroid tissues by immunohistochemistry, Western blot and RT–PCR

**Figure 2:**
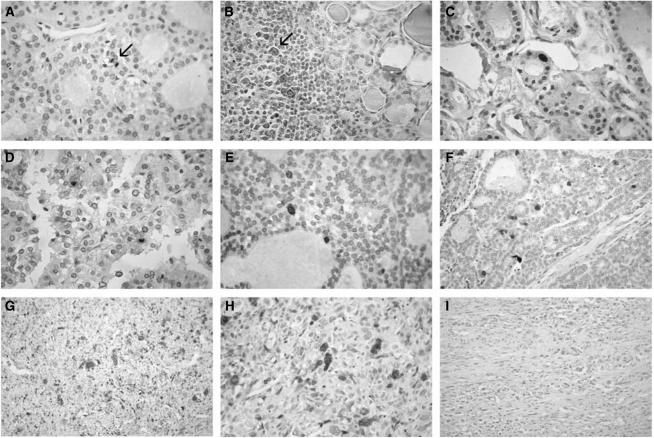
UbcH10 staining pattern in normal, inflammatory and neoplastic thyroid tissues. Follicular epithelial cells of normal thyroid (**A**) and oxyphilic cells of Hashimoto's thyroiditis (HT) (**B**) do not stain for UbcH10, with occasional mitotic figures (**A**, arrow) and lymphoid centroblasts of HT (**B**, arrow) providing the appropriate internal positive control. In neoplastic thyroid, UbcH10 staining pattern is strongly related to tumour grade, being weak in follicular adenoma (**C**), slightly more evident in well-differentiated papillary (**D**) and follicular (**E**) carcinomas, whereas stronger in poorly differentiated (**F**) and in anaplastic (**G**) carcinomas. In the latter, most of neoplastic cells show a very intense labelling, with intense nuclear staining (**H**), whereas signal disappeared by antigen incubation (**I**).

In order to evaluate whether the overexpression of UbcH10 is a feature of the thyroid tumours and not only of cultured thyroid carcinoma cell lines, we performed immunohistochemical analysis using a commercial antibody against UbcH10 protein. This methodology allows a rapid and sensitive screening of thyroid pathological tissues and is amenable to regular use as a routine diagnostic test. To find the best experimental conditions, ARO cell line and tumours, induced by injecting the ARO cell line into athymic mice, were used as positive controls (Cerutti *et al*, 1996). No staining was observed with normal human thyroid primary cell culture, whereas a positive staining was obtained with ARO cell line and ARO-induced tumours (data not shown). We found that normal thyroid, nodular goiter and Hashimoto's thyroiditis (HT) were almost always completely negative for UbcH10 expression. Only occasionally, single UbcH10-labelled thyroid epithelial cells showing mitotic figures could be observed by meticulous scrutiny (Figure 2A). In HT, there was a sharp contrast between the negative epithelial oxyphilic cells and the positive lymphoid germinal centers (Figure 2B). While a weak staining is detectable in follicular adenomas (Figure 2C), higher levels of UbcH10 were recorded in papillary (median value 2.2% of positive cells; range 0.9–4.1%), follicular (median value 2.8% of positive cells; range 1–6.1%) and poorly differentiated (median value 10.4% of positive cells; range 8–14.9%) carcinomas, signal being always easily detectable in the nuclei of scattered neoplastic cells (Figure 2D, E and F). UbcH10 staining pattern was somewhat different in anaplastic carcinomas, the percentage (median value 45.8% of positive cells; range 38.8–56.2%) of stained cells being large and the intensity of the neoplastic cells being strong (Figure 2G and H).

**Figure 3:**
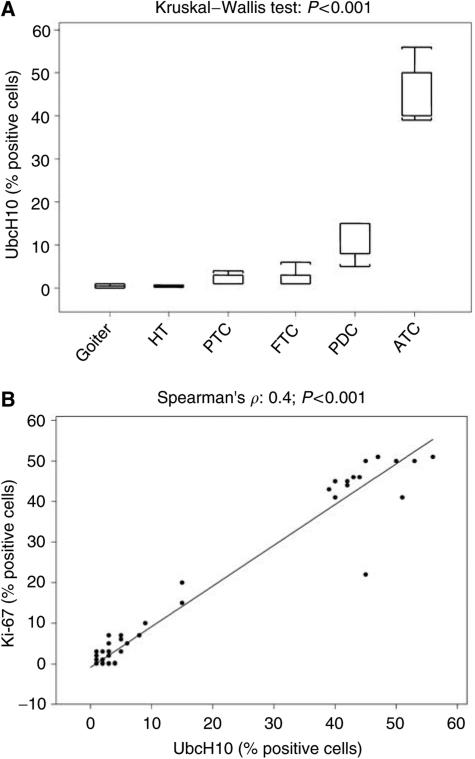
Statistical analysis of the immunohistochemical data. (**A**) Protein expression of UbcH10 (% positive cells) progressively increases in the several diagnostic categories from thyroid goiter to the thyroid anaplastic carcinomas. The analysis has been carried out using the Kruskal–Wallis test. HT, Hashimoto's thyroiditis; PTC, papillary thyroid carcinoma; FTC, follicular thyroid carcinoma; PDC, poorly differentiated thyroid carcinoma; ATC, anaplastic thyroid carcinoma. (**B**) Protein expression of UbcH10 (% positive cells) is correlated to that of Ki-67 (% positive cells) in the several diagnostic categories. The analysis has been carried out calculating the Spearman rank correlation coefficient.

No staining was observed when the same anaplastic carcinoma samples were stained with antibodies preincubated with UbcH10 recombinant protein (Figure 2I) or in the absence of the primary antibodies (data not shown). Therefore, as a general rule, UbcH10 expression is negligible in non-neoplastic thyroid, noticeable in well-differentiated carcinomas and conspicuous in less-differentiated tumours (Figure 3A).

To determine the relationship between UbcH10 expression and tissue proliferation, we correlated its expression in carcinomas with the proliferation rate of thyrocytes, as measured by Ki-67 staining; this latter showed the same tissue distribution of UbcH10, which was evident when adjacent (mirror) sections were stained. By using the Spearman rank order correlation, we determined that the association between UbcH10 and Ki-67 expression in thyroid cancer was statistically significant. In fact, the value of the Spearman *R* was 0.4 (*P*<0.001) (Figure 3B).

**Figure 4:**
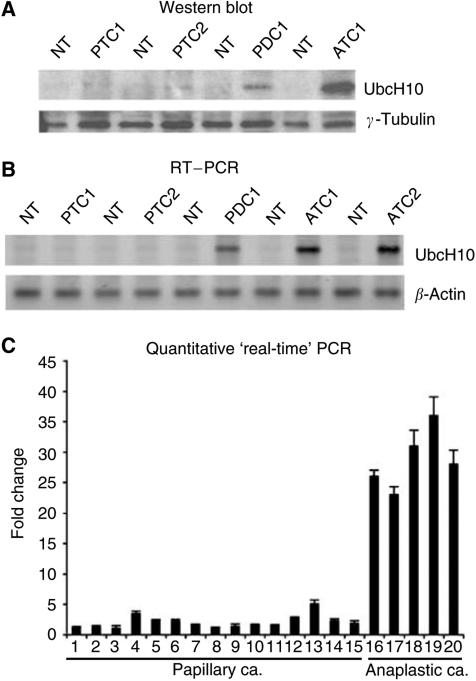
UbcH10 expression in human thyroid fresh tumour samples. (**A**) Western blot analysis of UbcH10 protein expression in a panel of thyroid neoplasias. The level of *γ*-tubulin has been used as loading control. NT, normal thyroid tissue; PTC1 and PTC2, papillary thyroid carcinomas from two different patients; PDC1, poorly differentiated carcinoma; ATC1, anaplastic thyroid carcinoma. (**B**) RT–PCR analysis of UbcH10 expression in human thyroid tumour samples *vs* their normal thyroid counterparts. *β*-Actin expression shows the same amount of RNAs used. NT, normal thyroid tissue; PTC1 and PTC2, papillary thyroid carcinomas from two different patients; PDC1, poorly differentiated carcinoma; ATC1 and ATC2, anaplastic thyroid carcinomas from two different patients. (**C**) Quantitative RT–PCR analysis was performed on human thyroid tumour samples of different histotype. The Fold Change values indicate the relative change in the expression levels between tumour samples and normal samples, assuming that the value of each normal sample is equal to 1.

Western blot analysis, performed on 30 surgically removed thyroid tumours, confirmed the immunohistochemical data. A representative Western blot is shown in Figure 4A. A strong band of 19.6 kDa corresponding to the UbcH10 protein was detected in anaplastic thyroid carcinomas and a weak one in poorly differentiated carcinomas, but not in papillary carcinomas and normal thyroids. These data strongly indicate that the expression of UbcH10 is more abundant in thyroid carcinomas characterised by a highly malignant and aggressive phenotype. Equal amounts of total proteins were used for each sample as demonstrated by the same gel analysed with an antibody against *γ*-tubulin.

UbcH10 expression was also evaluated by RT–PCR analysis on a panel of matched tumour/normal tissues. This analysis confirmed the protein data. In fact, as shown in Figure 4B, an amplified band of 115 bp was clearly detected in the anaplastic and poorly differentiated carcinoma samples, but not in the papillary ones and in all the corresponding normal thyroid tissues. Finally, quantitative RT–PCR analysis confirmed a great increase of UbcH10 expression in thyroid anaplastic samples, whereas a light increase was observed in papillary carcinoma samples (Figure 4C).

### UbcH10 expression in experimental models of thyroid carcinogenesis

**Figure 5:**
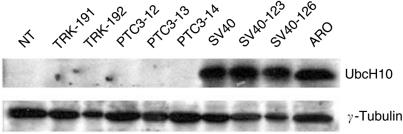
UbcH10 expression in experimental mouse thyroid tumours. Western blot analysis of experimental thyroid carcinomas developed in transgenic mice expressing TRK, RET-PTC-3 and large T SV40 oncogenes. ARO cell line was used as positive control. *γ*-Tubulin shows the same amount of protein level.

Thyroid neoplasias developing in transgenic animal lines expressing TRK (Tg-TRK) (Russell *et al*, 2000), RET/PTC3 (Tg-RET/PTC3) (Powell *et al*, 1998) and large T SV40 (Tg-SV40) (Ledent *et al*, 1991) oncogenes under the transcriptional control of the thyroglobulin promoter have been analysed for UbcH10 expression by Western blot analysis. Transgenic mice carrying TRK and RET/PTC3 oncogenes develop thyroid papillary carcinomas (Powell *et al*, 1998; Russell *et al*, 2000); thyroid anaplastic carcinomas were, conversely, obtained in the Tg-SV40 mice (Ledent *et al*, 1991). As shown in Figure 5, elevated UbcH10 protein levels were observed in the thyroid anaplastic carcinomas derived from large TSV40 transgenic mice. Conversely, UbcH10 protein was absent in normal mouse thyroid tissue and in the papillary carcinomas originating from TRK and RET/PTC3 mice.

Therefore, the analysis of the experimental models of thyroid carcinogenesis seems to confirm that the UbcH10 overexpression is essentially restricted to the undifferentiated histotype.

### Suppression of the UbcH10 synthesis inhibits thyroid carcinoma cell growth

**Figure 6:**
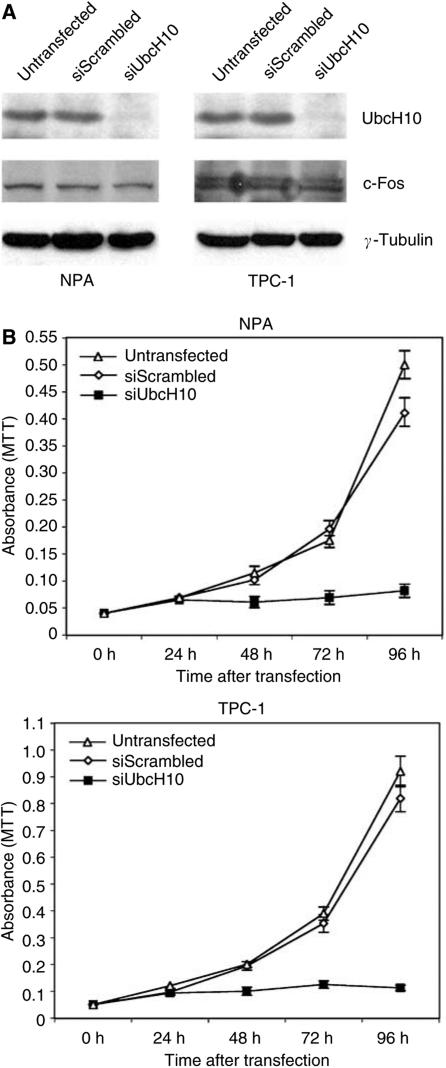
The block of UbcH10 protein synthesis by RNA interference inhibits the proliferation of thyroid carcinoma cells. (**A**) Inhibition of UbcH10 protein expression by RNAi in NPA and TPC-1 cell lines evaluated by Western blot analysis. At 48 h after siRNA transfection, total cell lysates were prepared and normalised for protein concentration. The expression of *γ*-tubulin was used to control equal protein loading (30 *μ*g). In this figure, we also show the expression of a fast turning-over gene like c-Fos to confirm the specific effect of siRNAs against UbcH10. (**B**) Growth curves of NPA and TPC-1 cell lines after siUbcH10 treatment. NPA and TPC-1 cells were transfected with siUbcH10 duplexes (siUbcH10) and the relative number of viable cells was determined by MTT assay. Cells transfected with a scrambled duplex (siScrambled) and untransfected cells (Untransfected) were used as negative controls. Absorbance was read at 570 nm and the data are the mean of triplicates.

We asked whether UbcH10 overexpression had a role in the process of thyroid carcinogenesis by evaluating the growth rate of two thyroid carcinoma cell lines, in which UbcH10 protein was suppressed by RNA interference. The NPA and TPC-1 cell lines were treated with siRNA duplexes targeting to the UbcH10 mRNA. After transfection, we observed an efficient knockdown of the UbcH10 protein levels at 48 h after treatment (Figure 6A). The analysis of cell growth of these cell lines in the presence or absence of the UbcH10 siRNA duplexes revealed that the block of the UbcH10 protein synthesis significantly inhibits thyroid carcinoma cell growth. In fact, as shown in Figure 6B, a significant reduction in cell growth rate was observed in NPA and TPC-1 cell lines treated with UbcH10 siRNA in comparison to the untreated cells or those treated with the control scrambled siRNA.

These results indicate a role of UbcH10 in neoplastic thyroid cell proliferation.

### UbcH10 overexpression is not sufficient to transform rat thyroid cells

To further characterise the role of UbcH10 in thyroid carcinogenesis, we transfected normal rat thyroid cells with an expression vector carrying the UbcH10 gene under the transcriptional control of the cytomegalovirus promoter. The selected clones were shown to express high UbcH10 protein levels (data not shown). We evaluated the growth rate of the UbcH10-transfected PC Cl 3 cells and the same cells transfected with a backbone vector: no differences were observed. Equally, the neoplastic phenotype of the UbcH10-transfected PC Cl 3 cells was evaluated by a soft agar colony assay and by injection into athymic mice. As reported in Table 1, the PC Cl 3 cells transfected with the UbcH10 expression vector were not able to give rise to colonies in soft agar and induce tumours in athymic mice. As a positive control, we used the PC Cl 3 cells transformed with the Myeloproliferative sarcoma virus (PC MPSV): these cells have a very highly malignant phenotype (Fusco *et al*, 1987).

These results indicate that UbcH10 overexpression is not able to transform rat thyroid cells *in vitro*.

## DISCUSSION

Thyroid neoplasms represent an excellent model for studying the process of cell transformation since they include a broad spectrum of histotypes showing different degree of malignancy (Hedinger *et al*, 1989; Wynford-Thomas, 1997). From the Affymetrix microarray analysis, we found the UbcH10 gene that appeared greatly increased in all of the thyroid carcinoma cell lines. UbcH10 was previously identified as a human homologue of the cyclin-selective E2 (E2-c), which is required for the destruction of mitotic cyclins by the ubiquitination pathway. The UbcH10 gene belongs to the E2 gene family and codes for a protein of 19.6 kDa that is involved in the ubiquitin-dependent proteolysis. In this system, three distinct enzymes cooperate to process target proteins for degradation. More precisely, the ubiquitin-conjugating enzyme (E2) transfers activated ubiquitin by ubiquitin-activating enzyme (E1) to a lysine residue of the target proteins in cooperation with the ubiquitin-ligase (E3). Polyubiquitinated proteins are then recognised by the 26S proteasome and rapidly degraded (Hershko and Ciechanover, 1998; Joazeiro and Weissman, 2000).

In our study, we have evaluated the expression of UbcH10 in human thyroid neoplasias and in mouse experimental tumours. No significant UbcH10 expression was observed in normal thyroids, goiters and adenomas, whereas a great induction of UbcH10 expression was observed in anaplastic human thyroid carcinomas and in experimental undifferentiated thyroid tumours. Just a weak expression of UbcH10 was observed in follicular and papillary human thyroid carcinomas. Therefore, these data strongly indicate that UbcH10 overexpression could be associated with the thyroid tumour progression since there is a good correlation with the late stage of thyroid neoplastic transformation. The low UbcH10 levels detected in the differentiated thyroid malignancies would appear in contrast with the data showing an abundant UbcH10 expression in the cell lines deriving from differentiated carcinomas. We retain this discrepancy only apparent since thyroid carcinoma cell lines, even deriving from differentiated tumours, cannot be completely compared to surgically removed tumours. In fact, these cell lines harbour p53 mutations that are rare in thyroid differentiated neoplasias (Tanaka *et al*, 1987; Estour *et al*, 1989; Pang *et al*, 1989; Ito *et al*, 1992; Dobashi *et al*, 1993; Donghi *et al*, 1993; Fagin *et al*, 1993; Zeki *et al*, 1993; Fabien *et al*, 1994; Matias-Guiu *et al*, 1994; Ain *et al*, 1997; Fiore *et al*, 1997; Basolo *et al*, 2002) and they have a high proliferation rate. However, this consideration does not exclude the validity of the use of the thyroid carcinoma cell lines as experimental model to draw new information that, however, need to be subsequently validated on fresh tumours.

Our results also indicate a correlation between UbcH10 overexpression and the proliferation status since there is a good association with the proliferation marker Ki-67/MIB1.

An aim of our work was to evaluate the possible causal role of UbcH10 overexpression in thyroid carcinogenesis. Indeed, the block of protein synthesis significantly inhibited the growth of several thyroid carcinoma cell lines, suggesting an important role of UbcH10 in thyroid cell proliferation, and then in the progression step of thyroid carcinogenesis.

Therefore, even though the mechanisms by which UbcH10 overproduction contributes to the neoplastic phenotype remains unclear, we can assume that it leads to a deregulation of cell growth. These results are quite consistent with previous published data showing that UbcH10 was expressed at high levels in primary tumours derived from the lung, stomach, uterus, and bladder as compared with their corresponding normal tissues, suggesting that UbcH10 is involved in tumorigenesis or cancer progression (Okamoto *et al*, 2003; Wagner *et al*, 2004). It has also been shown that UbcH10 is upregulated in NIH3T3 cell line transformed by EWS/FLI1, but not in untransformed NIH3T3 cell clone expressing EWS/FLI1 (Arvand *et al*, 1998). Moreover, it has been shown that UbcH10 is required for override metaphase, likely degrading growth suppressor, and for destruction of mitotic cyclins, indicating a role of UbcH10 in cell cycle progression (Arvand *et al*, 1998).

However, overexpression of UbcH10 in normal rat thyroid cells did not affect cell growth neither induced a malignant phenotype, indicating that UbcH10 overexpression is not sufficient for malignant thyroid cell transformation. This result might appear in contrast with those showing that NIH3T3 stable transfectants overexpressing UbcH10 exhibited a malignant phenotype as compared with parental NIH3T3 cells (Okamoto *et al*, 2003). This discrepancy is, in our opinion, just apparent since we have to consider that NIH 3T3 cells are preneoplastic cells, whereas PC Cl 3 cells are much more resistant to express the neoplastic phenotype, since even the expression of several oncogenes (v-ras-Ki, v-ras-Ha, etc.) are not able to lead these cells to the fully malignant phenotype that is achieved only when there is a synergy of two different oncogenes (Fusco *et al*, 1987). In conclusion, our data propose the UbcH10 overexpression as a feature of the anaplastic carcinoma histotype. The block of UbcH10 expression significantly reduced the growth of thyroid carcinoma cell lines indicating an involvement of UbcH10 in the increased proliferation of these carcinoma cell lines. Therefore, these results open the perspective of a therapy of the anaplastic thyroid carcinoma, one of the most aggressive tumours in mankind, based on the suppression of the UbcH10 synthesis and/or function.

## Figures and Tables

**Table 1:** Analysis of the neoplastic phenotype of the UbcH10-transfected rat thyroid cell lines

**Cell line**	**Doubling time (h)**	**Colony-forming^a^ efficiency (%)**	**Tumour^b^ incidence**
PC CL 3	24	0	0/4
PC UbcH10 CL1	24	0	0/4
PC UbcH10 CL2	23	0	0/4
PC MPSV	18	70	4/4

a Colony-forming efficiency was calculated by the formula (number of colonies formed/number of plated cells) × 100.

b Tumorigenicity was assayed by injecting 2 × 10^6^ cells into athymic mice (4–6 weeks old).
